# Inhibition of lysophosphatidic acid receptor 1–3 deteriorates experimental autoimmune encephalomyelitis by inducing oxidative stress

**DOI:** 10.1186/s12974-021-02278-w

**Published:** 2021-10-19

**Authors:** Jong Hee Choi, Jinhee Oh, Min Jung Lee, Hyunsu Bae, Seong-Gyu Ko, Seung-Yeol Nah, Ik-Hyun Cho

**Affiliations:** 1 Department of Convergence Korean Medical Science, College of Korean Medicine, Kyung Hee University, Seoul, 02447 Republic of Korea; 2 Department of Physiology, College of Korean Medicine, Kyung Hee University, Seoul, 02447 Republic of Korea; 3 Korean Medicine-Based Drug Repositioning Cancer Research Center, College of Korean Medicine, Kyung Hee University, Seoul, 02447 Republic of Korea; 4 Ginsentology Research Laboratory and Department of Physiology, College of Veterinary Medicine and Bio/Molecular Informatics Center, Konkuk University, Seoul, 05029 Republic of Korea; 5 Institute of Convergence Korean Medicine, Kyung Hee University, Seoul, 02447 Republic of Korea

**Keywords:** Lysophosphatidic acid receptors, Experimental autoimmune encephalomyelitis, Reactive oxygen species, NADPH oxidase

## Abstract

**Background:**

Lysophosphatidic acid receptors (LPARs) are G-protein-coupled receptors involved in many physiological functions in the central nervous system. However, the role of the LPARs in multiple sclerosis (MS) has not been clearly defined yet.

**Methods:**

Here, we investigated the roles of LPARs in myelin oligodendrocyte glycoprotein peptides-induced experimental autoimmune encephalomyelitis (EAE), an animal model of MS.

**Results:**

Pre-inhibition with LPAR1–3 antagonist Ki16425 deteriorated motor disability of EAE^low^. Specifically, LPAR1–3 antagonist (intraperitoneal) deteriorated symptoms of EAE^low^ associated with increased demyelination, chemokine expression, cellular infiltration, and immune cell activation (microglia and macrophage) in spinal cords of mice compared to the sham group. This LPAR1–3 antagonist also increased the infiltration of CD4^+^/IFN-γ^+^ (Th1) and CD4^+^/IL-17^+^ (Th17) cells into spinal cords of EAE^low^ mice along with upregulated mRNA expression of IFN-γ and IL-17 and impaired blood–brain barrier (BBB) in the spinal cord. The underlying mechanism for negative effects of LPAR1–3 antagonist was associated with the overproduction of reactive oxygen species (ROS)-generating nicotinamide adenine dinucleotide phosphate (NADPH) oxidases (NOX) 2 and NOX3. Interestingly, LPAR1/2 agonist 1-oleoyl-LPA (LPA 18:1) (intraperitoneal) ameliorated symptoms of EAE^high^ and improved representative pathological features of spinal cords of EAE^high^ mice.

**Conclusions:**

Our findings strongly suggest that some agents that can stimulate LPARs might have potential therapeutic implications for autoimmune demyelinating diseases such as MS.

## Background

Multiple sclerosis (MS) is a chronic, inflammatory, autoimmune, and demyelinating disease of the central nervous system (CNS). Specific symptoms of MS can include double vision, blindness in one eye, muscle weakness, trouble with sensation, stiffness, and spasms. While the exact etiology of MS is unclear, it is thought to be due to a combination of genetic and environmental factors such as infectious agents [1–3]. The pathology of MS is related to demyelination, axonal and oligodendroglial loss, reactive astrocytes or gliotic scar formation, disruption of the blood–brain barrier (BBB), and infiltration of peripheral immune cells, including lymphocytes and macrophages [4, 5]. Unfortunately, complete cure for MS is currently unknown. MS patients usually take intravenous steroids, anti-inflammatory medications (corticosteroids), disease-modifying drugs, and so on, to improve their function after an attack and prevent new attacks [2, 3, 6]. However, these treatments have limited efficacy. In addition, adverse drug reactions such as irritation at the injection site, influenza-like syndrome, and heart palpitations might occur during long-term medication [3, 6]. Therefore, it is essential to research and develop innovative medications for delaying the onset of MS or forestalling its progression.

Oxidative stress is characterized as an imbalance between the production of reactive oxygen species (ROS) and the antioxidant capacity of the cell [7, 8]. ROS can induce mitochondrial DNA mutations, damage the mitochondrial respiratory chain, alter membrane permeability, and influence intracellular Ca^2+^ homeostasis and mitochondrial defense systems [7, 8]. Normally, ROS are generated by nicotinamide adenine dinucleotide phosphate (NADPH) oxidases (NOX) and eliminated from cells by reducing agents or by enzymatic reactions to maintain homeostasis in the body [9]. ROS at low physiological intracellular levels can promote cellular growth and survival signaling pathways, whereas ROS at higher levels can induce growth arrest and cellular apoptosis or senescence. Interestingly, excessive ROS play a crucial role in various pathologic mechanisms underlying MS and EAE [7, 8]. In the early stage of lesion (demyelination) formation, ROS may lead to BBB disruption and accelerate transendothelial migration of peripheral immune cells such as T cells and macrophages into the CNS [7, 8]. Consequently, ROS play a critical role in lesion persistence or deterioration in MS and EAE by continuing worsening demyelination and inducing axonal and oligodendrocyte damage [7, 8]. Thus, antioxidants that can inhibit excessive generation of ROS or escape harmful activities of ROS might be good therapeutics for preventing and treating MS and EAE [7, 8]. Currently, accumulated evidences have suggested that ROS are generated by lysophosphatidic acid (LPA) signaling in adipose derived stem cells [10], PC-3 human prostate cancer cells [11], ovarian cancer cells [12], and mouse J774A.1 macrophages [13]. These reports strongly suggest that regulating LPA signaling might influence the pathologic mechanism underlying MS and EAE.

LPA is a major lysophospholipid that acts as both a minor membrane component and an extracellular signaling mediator in numerous organs, tissues, and body fluids [14]. LPA primarily signals via the activation of six cognate G protein-coupled receptors, lysophosphatidic acid receptors (LPARs) 1–5, and atypical LPAR6 [14]. LPARs are differentially expressed in most cell types of central and peripheral nervous tissues. They are involved in many functions of neuronal networks [14]. LPA signaling via LPARs can influence cell survival, cell differentiation, cell proliferation, cell migration, angiogenesis, neurogenesis, and neuroplasticity in normal and abnormal nervous systems [14]. It can stimulate chemotaxis, polarization, motility, and transendothelial migration of immature murine dendritic cells or naive T cells of the immune system into the CNS [15–17]. Interestingly, serum levels of LPAs in MS patients and EAE murine model are decreased compared to those in healthy controls whereas those of LPAs in spinal cords in T cell receptor transgenic mice (relapsing–remitting mice) are increased during symptom-free and recovery intervals of experimental autoimmune encephalomyelitis (EAE) model, a murine model mimicking MS [18]. LPAR2-positive T cells and myeloid cells are diminished in the spleen of EAE mice while its mRNA levels are increased in circulating white blood cells and lumbar spinal cords of EAE mice [18]. These reports strongly suggest that LPARs might be crucial receptors in the pathogenesis of MS and EAE. Although complexities of LPA receptor signaling in neuronal inflammation such as MS and EAE have been demonstrated, they are not well appreciated yet. Here, we explored whether LPAR1–3 antagonist or agonist could regulate motor disability and inflammation in an EAE murine model. Our findings suggest that some agonists for LPAR1–3 might be useful as therapeutics to prevent and treat autoimmune demyelinating diseases such as MS.

## Methods

### Experimental animals and ethic approval

The 8- to 9-week-old C57BL/6J female mice (weight, 19–21 g) were purchased from the Narabiotec Co., Ltd. (Seoul, Republic of Korea). Their seed mice originated from Taconic Biosciences Inc. (Cambridge, IN, USA). The mice were allowed free access to the usual standard laboratory food and tap water. The mice were housed under a 12-h light/dark cycle (light on 08:30–20:30) at room temperature (23 ± 2 °C) and humidity (54 ± 15%). Animal experiments were approved by the Institutional Animal Care and Use Committee (IACUC) of Kyung Hee University (KHUASP-18-174). Animal treatment and maintenance were carried out in accordance with IACUC guidelines. In this process, proper randomization of laboratory animals and handling of data were performed in a blinded manner in accordance with recent recommendations from an NIH workshop on preclinical models of neurological diseases [19].

### Experimental group and drug treatment

To investigate the effect of Ki16425, LPAR1–3 antagonist, on EAE^low^, the experimental group was randomly divided into the following groups (*n* = 5 per group): the Sham [vehicle treatment, s.c. + saline, i.p.], EAE^low^ [200 µg of myelin oligodendrocyte glycoprotein (MOG)_35–55_ peptide, s.c. + saline, i.p.], EAE^low^ + Ki16425 [200 µg of MOG_35–55_, s.c. + 15 and 30 mg/kg of Ki16425, i.p.], and Ki16425 alone groups [vehicle treatment, s.c. + 30 mg/kg of Ki16425, i.p.]. Ki16425, an LPAR1–3 antagonist (Tocris Bioscience, Bristol, UK), was prepared in 5% dimethyl sulfoxide (DMSO)/phosphate buffered saline (PBS) and was administered once daily from onset phase (day 9 after EAE^low^ induction; mean score of motor disability, approximately 0.5). To examine the effect of 1-oleoyl-LPA (LPA 18:1), LPAR1/2 agonist, on EAE^high^, the experimental group was randomly divided into the following groups (*n* = 5 per group): the Sham [vehicle treatment, s.c. + saline, i.p.], EAE^high^ [200 µg of MOG_35–55_, s.c. + saline, i.p.], EAE^high^ + 1-oleoyl-LPA [200 µg of MOG_35–55_, s.c. + 0.5 and 1 mg/kg of 1-oleoyl-LPA, i.p.], and LPA alone group [vehicle treatment, s.c. + 1.0 mg/kg of 1-oleoyl-LPA, i.p.]. 1-oleoyl-LPA, an LPAR1/2 agonist was dissolved in PBS and administered once daily for 10 days from onset phase (day 8 after EAE^high^ induction; mean score of motor disability, approximately 0.5). The same experiments were repeated 3 times.

### EAE induction and motor disability assessment

Mice were immunized with 100 μl of emulsion containing MOG_35–55_ peptide (Sigma, St. Louis, MO, USA) in PBS, 100 μl of complete Freund’s adjuvant (CFA; Sigma), and *Mycobacterium tuberculosis* extract H37Ra (Mtb) into the hind flank subcutaneously. Mice also received 200 ng of pertussis toxin (Sigma, St. Louis, MO, USA) through i.p. injection on the day of immunization and at 48 h after immunization. EAE was induced by two different methods. The EAE model presenting low scores of motor disability (called EAE^low^) was induced with 200 µg of MOG peptide and 300 µg of Mtb. The EAE model presenting high scores of motor disability (called EAE^high^) was induced with 200 µg of MOG peptide and 500 µg of Mtb. These models were used to investigate effects of Ki16425 (LPAR1–3 antagonist) and 1-oleoyl-LPA (LPAR1/2 agonist) on EAE. The same experiments were repeated 3 times. After immunization, mice were observed daily to record scores of motor disability using the following criteria: score 0, no signs; score 1, mild loss of tail tone; score 2, complete loss of tail tone without hind limb paralysis; score 3, complete loss of tail tone and hind limb weakness (abnormal gait); score 4, complete hind limb paralysis; score 5, complete hind limb paralysis and forelimb weakness (or unilateral forelimb paralysis); score 6, quadriplegia, moribund condition; and score; 7, death [20–22].

### Cryosections preparation and histopathological staining

At the peak stage (19–20 days) of neurological symptoms after EAE^low^ and EAE^high^ induction, mice (*n* = 5 per group) were euthanized under diethyl ether anesthesia and perfused intracardially with 0.9% saline followed by 4% paraformaldehyde in 0.1 M phosphate buffer (pH 7.4). Lumbar spinal cords were removed and cryosections (10 μm thick; *n* = 3 per spinal cord; 5 spinal cords per group) were prepared as previously described [20–22]. The cryosections were stained with luxol fast blue (LFB) dye and hematoxylin and eosin (H&E) to evaluate demyelination and immune cell infiltration, respectively, as previously described [20–22].

### Quantification of demyelination and cellular infiltration

To quantify the level of demyelination after LFB staining, demyelinated area and total areas of white matter were measured for three fields per section (3 sections per spinal cord, 5 spinal cords per group) using ImageJ Software (NIH, USA). The final % value for demyelination was presented as mean demyelinated area (μm^2^) per optical field (at 200× magnification). The level of cellular infiltration after H&E staining was quantified by semi-manually counting the number of total inflammatory cells per optical field using the ImageJ Software. For each group, three fields per section (3 sections per spinal cord, 5 spinal cords per group) were analyzed. The final value was presented as total cell number per optical field. The investigators who performed these tissue investigations were blinded to mouse groups until the end of the experiment after graphs were generated and tissue sections were compared.

### Immunofluorescence and immunohistochemistry evaluation

Immunofluorescence analysis was accomplished as previously described [20–22]. Briefly, cryosections (10 μm thick; *n* = 3 per spinal cord; 5 spinal cords per group) from lumbar spinal cords in each group were incubated with rat anti-CD3 (1:500; BD Biosciences, NJ, USA), rat anti-myelin basic protein (MBP) (1:1000; Abcam), anti-ionized calcium binding adaptor molecule-1 (Iba-1) antibody (1:2000; Wako, Osaka, Japan), mouse anti-glial fibrillary acidic protein (GFAP; 1:2000; Santa Cruz Biotechnology, Santa Cruz, CA, USA) and/or rat anti-platelet endothelial cell adhesion molecule (PECAM)-1 (1:500; Santa cruz), mouse anti-albumin (1:500; Santa cruz), and rabbit anti-immunoglobulin G (IgG) (1:500; Abcam), mouse anti-occludin (1:500; Invitrogen, MA, USA), mouse anti-ZO-1 (1:500; Invitrogen) as primary antiserum and cyanine 3- and fluorescein-isothiocyanate (FITC)-conjugated mouse/rabbit/rat IgG antibody (1:200–1:500; Jackson ImmunoResearch, West Grove, PA, USA) as secondary antiserum. Images from each section were captured using confocal imaging system (LSM 5 PASCAL; Carl Zeiss, Oberkochen, Germany) and their intensity quantified. Immunohistochemical analysis for Iba-1 was accomplished and analyzed as previously described [20–22].

### Western blot assessment

At the peak stage (19–20 days) of neurological symptoms after EAE^low^ and EAE^high^ induction, the lumbar segments of the spinal cords (*n* = 3 per group) from each group were rapidly cropped under diethyl ether anesthesia. Western blot analysis was performed by previous described [20–22] using rat anti-CD3 (1:500; BD Biosciences), rabbit anti-LPAR1–3 (1:1000; Abcam), rat anti-MBP (1:1000; Abcam), rabbit anti-Iba-1 (1:1000; Wako), rat anti-PECAM-1 (1:500; Santa cruz), mouse anti-GFAP (1:2000; Millipore), rabbit anti-cyclooxygenase (COX)-2 (1:1000; Santa Cruz), rabbit anti-phospho (p)-nuclear factor-kappa B (NF-κB) NF-κB, p-p38, and p38 (1:1000; Cell signaling technology), goat anti-4*-*hydroxynonenal (4-HNE) (1:500; Abcam), or horseradish peroxidase-conjugated secondary antibodies (1:200; Vector Laboratories, Burlingame, CA, USA). Anti-glyceraldehyde-3-phosphate dehydrogenase (GAPDH) antibody (1:1000; Cell Signaling Technology) was used as an internal control for relative protein quantification. Polyvinylidene fluoride membranes were visualized using a super cooled-CCD camera system with a Davinch-K Gel imaging system (Dvinch-K, Seoul, South Korea). The density of each band was converted to numerical values using NIH Image J program (http://rsbweb.nih.gov/ij/), with the background values subtracted from an area of film immediately adjacent to the stained band. Data are expressed as the ratio of each value against GAPDH for each sample. Experiments were repeated three times with similar protocol.

### Real-time polymerase chain reaction (PCR) analyses

Real-time PCR was accomplished using SYBR Green PCR Master Mix (ABI, Warrington, UK) as described previously described [20–22]. Reactions (*n* = 3 per group) were performed in duplicate in a total volume of 10 μl containing 10 pM primer, 4 μl cDNA, and 5 μl SYBR Green PCR Master Mix. The mRNA levels of each target gene were normalized to that of GAPDH mRNA. Fold-induction was calculated using the 2^−∆∆CT^ method, as previously described [23]. All real-time RT-PCR experiments were performed in triplicates, repeated at least three times. The values are presented as mean ± SEM values unless otherwise noted. The primer sequences were as follows: monocyte chemoattractant protein (MCP)-1–5′-CTT CTG GGC CTG CTG TTC A-3′ and 5′-CCA GCC TAC TCA TTG GGA TCA-3′, macrophage inflammatory protein (MIP)-1α-5′-CAG CCA GGT GTC ATT TTC CT-3′ and 5′-AGG CAT TCA GTT CCA GGT CA-3′, regulated upon activation, normal T cell expressed and presumably secreted (RANTES)-5′-ACA CCA CTC CCT GCT GCT TT-3′ and 5′-GAC TGC AAG ATT GGA GCA CTT GA-3′, CD3-5′-CTC TGG GCT TGC TGA TGG-3′ and 5′-GGT TGG GAA CAG GTG GTG-3′, T-bet-5′-CGG AGC GGA CCA ACA GCA TCG TTT C-3′ and 5′-CAG GGT AGC CAT CCA CGG GCG GGT-3′, interferon-gamma (IFN)-γ-5′-ACA ATG AAC GCT ACA CAC TGC AT-3′ and 5′-TGG CAG TAA CAG CCA GAA ACA-3′, ROR-γt-5′-ACC TCT TTT CAC GGG AGG A-3′ and 5′-TCC CAC ATC TCC CAC ATT G-3′, interleukin (IL)-17A-5′-GTG TCT CTG ATG CTG TTG-3′ and 5′-AAC GGT TGA GGT AGT CTG-3′, Foxp3-5′-GGC CCT TCT CCA GGA CAG A-3′ and 5′-GCT GAT CAT GGC TGG GTT GT-3′, transforming growth factor (TGF)-ß-5′-GCC CTG GAT ACC AAC TAT TGC-3′ and 5′-GCA GGA GCG CAC AAT CAT GTT-3′, GATA3-5′-GAA GGC ATC CAG ACC CGA AAC-3′ and 5′-ACC CAT GGC GGT GAC CAT GC-3′, IL-4–5′-CGA AGA ACA CCA CAG AGA GTG AGC T-3′ and 5′-GAC TCA TTC ATG GTG CAG CTT ATC G-3′, occludin-5′-ATG CAT CTC TCC GCC ATA CAT-3′ and 5′-AGA CCT GAT GAA TTC AAA CCC AAT-3, claudin-5–5′-ACG GGA GGA GCG CTT TAC3′ and 5′-GTT GGC GAA CCA GCA GAG-3, intercellular adhesion molecule (ICAM)-1–5′-TGC GTT TTG GAG CTA GCG GAC CA-3′ and 5′-CGA GGA CCA TAC AGC ACG TGC AG-3′, vascular cell adhesion molecule (VCAM)-1–5′-CCT CAC TTG CAG CAC TAC GGG CT-3′ and 5′-TTT TCC AAT ATC CTC AAT GAC GGG-3′, cyclooxygenase (COX)-2–5′-CAG TAT CAG AAC CGC ATT GCC-3′ and 5′-GAG CAA GTC CGT GTT CAA GGA-3′, iNOS-5′-GGC AAA CCC AAG GTC TAG GTT-3′ and 5′-TCG CTC AAG TTC AGC TTG GT-3′, tumor necrosis factor (TNF)-α-5′-AGC AAA CCA CCA AGT GGA GGA-3′ and 5′-GCT GGC ACC ACT AGT TGG TTG T-3′, NAD(P)H: quinone oxidoreductase (NOX)1–5′-AGG TCG TGA TTA CCA AGG TTG TC-3′ and 5′-AAG CCT CGC TTC CTC ATC TG-3′, NOX2-5′-ACT CCT TGG GTC AGC ACT GG-3′ and 5′-GTT CCT GTC CAG TTG TCT TCG-3′, NOX3-5′-GTG ATA ACA GGC TTA AAG CAG AAG GC-3′ and 5′-CCA CTT TCC CCT ACT TGA CTT TAG-3′, NOX4-5′-CCT CAT GGT TAG AGC TTC TAC CTA CGC-3′ and 5′- TGA CTG AGG TAC AGC TGG ATG TTC AC-3′, and GAPDH-5′-AGG TCA TCC CAG AGC TGA ACG-3′ and 5′-CAC CCT GTT GCT GTA GCC GTA T-3′.

### Measurement of weights of spleen and lymph nodes

At the peak day (19–20 days after EAE^low^ induction) of neurological disorder, 5 mice in each group were anesthetized. Their spleens and lymph nodes were carefully removed without fat, connective tissue, or fluid. They were weighed using a microbalance (OHAUS, Parsippany, USA). The experiment was repeated three times.

### Flow cytometry

At the peak stage of neurological impairment (19–20 days after EAE^low^ induction), 5 mice from each group were euthanized under brief diethyl ether anesthesia followed by perfusion with 0.9% physiological saline and then spleen and lumbar spinal cord were carefully cropped. To measure the level of cell population, single-cell suspensions refined from whole tissue were prepared as previously described [20–22]. For surface cell analysis, single-cell suspensions were incubated with APC anti-mouse CD11b (OX-42; Biolegend, San Diego, CA, USA), PE anti-mouse CD45 (OX-1; Biolegend), APC anti-mouse CD4 (OX-35, BD Biosciences), and PE anti-mouse CD8a (OX-8, BD Biosciences) for 30 min at 4 °C. Microglia and macrophages were identified based on their relative CD45 expression levels [20–22, 24]. Briefly, after acquiring unstained and single colored control samples to calculate compensation matrix, we acquired 1 × 10^4^ events within the combined gate based on physical parameters [forward scatter (FSC) and side scatter (SSC)]. CD11b^+^/CD45^+(low)^ cells and CD11b^+^/CD45^+(high)^ cells were gated as resident microglia and macrophages, respectively. For intracellular cell analysis, cells were restimulated with phorbol-12-myristate-13-acetate and ionomycin and Golgistop in RPMI media. After 5 h, cells were stained with PerCP-Cy 5.5 anti-mouse CD4 (RM4-5; BD Biosciences), FITC anti-mouse IFN-γ (XMG1.2; BD Biosciences), PE anti-mouse t-bet (4B10; BD Biosciences), PE anti-mouse IL-17A (TC11-18H10; BD Biosciences), APC anti-mouse RORγt (B2D; eBioscience, NH, USA), PE anti-mouse IL-4 (11B11; BD Biosciences), PE anti-mouse CD25 (PC61.5; FJK-16 s; eBioscience), and APC anti-mouse/rat Foxp3 (FJK-16 s; eBioscience) for 30 min at 4 °C. The cells were washed twice with 2% fetal bovine serum in PBS and used for flow cytometry as described [20–22]. To identify CD4^+^ T cell populations, cells (1 × 10^4^) were first gated using FSC and SSC properties. CD4^+^ T cells were used to analyze populations of Th1 (CD4^+^/IFN-γ^+^/T-bet^+^), Th17 (CD4^+^/IL-17A^+^/RORγt^+^), Treg (CD4^+^/CD25^+^/Foxp3^+^), and Th2 (CD4^+^/IL-4^+^) cells on CD4^+^ T cells (Fig. 4J). Three-color staining of one cell was performed for simultaneous analysis. Intracellular cytokine levels were indicated as percentages within CD4^+^ population. Data were collected on a FACS Calibur flow cytometer (BD Biosciences) and analyzed using Cell Quest Pro software (BD Biosciences) described [20–22]. Experiments were repeated three times with same protocol.

### Statistical analyses

Statistical analysis was performed using the SPSS 24.0 package (SPSS Inc, Chicago, USA) for Windows. Neurological scores obtained by EAE induction were analyzed using two-way analysis of variance (ANOVA) with repeated measures with one within-subjects factor (time) and two between-subject factors (Sham and EAE group; EAE and EAE + antagonist/or agonist group). The data from organ weight, immunohistochemistry, Western blot, flow cytometry, and real-time PCR analysis were performed using one-way ANOVA with Tukey post hoc test for comparison of multiple groups. The data were presented as mean ± SEM. *p* values of less than 0.05 were accepted as statistically significant.

## Results

### Ki16425 deteriorates motor disability and spinal demyelination during EAE^low^

**Fig. 1:**
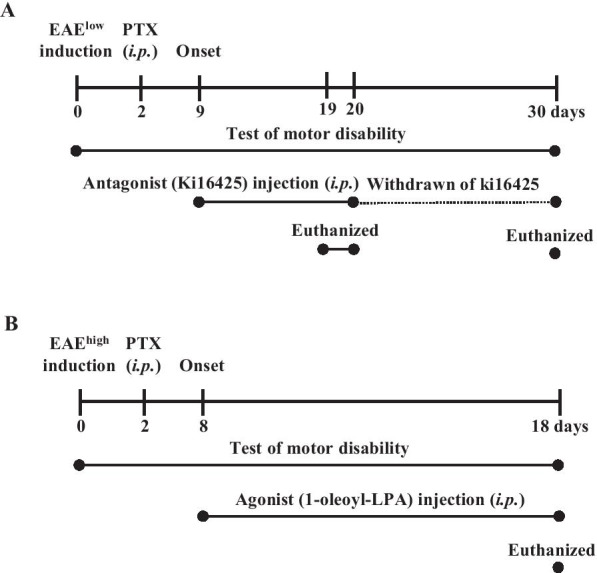
Schematic design of the experimental protocol. **A**, **B** EAE^low^ mice were C57BL/6J mice s.c. induced by immunization with 200 µg of MOG_35–55_ and 300 µg of Mtb (**A**), while EAE^high^ mice were induced with 200 µg of MOG peptide and 500 µg of Mtb (**B**). LPAR1–3 antagonist (**A**) or agonist (**B**) was i.p. injected once daily from onset phase (day 8–9 after EAE induction) of motor disability. Level of motor disability was then measured daily until the last day of the experiment. For various analyses, mice were euthanized at the peak phase (day 19–20 after EAE induction) of neurological symptoms

**Fig. 2:**
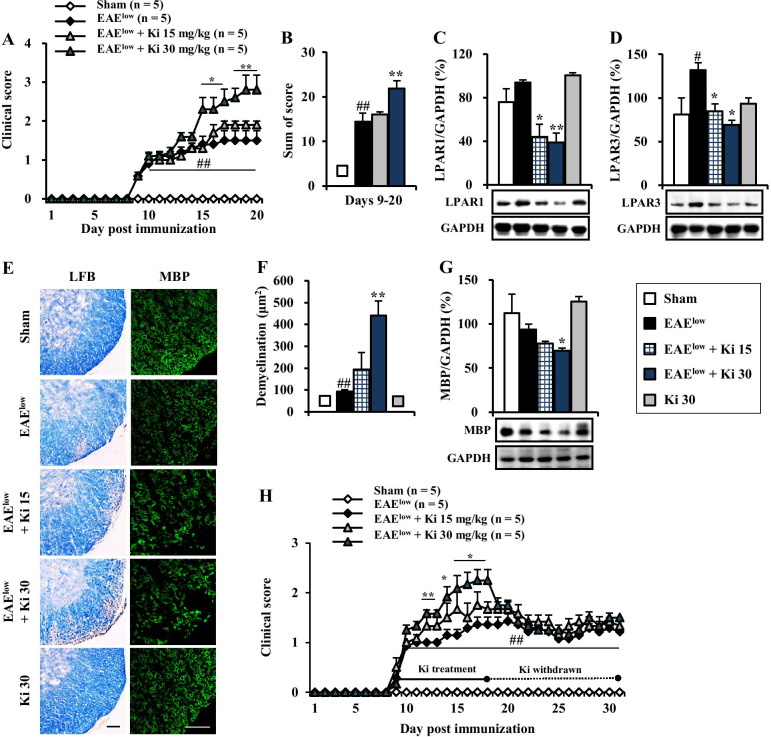
Effects of Ki16425 on motor disability and spinal demyelination during EAE^low^. **A**, **B** Following immunization, neurological symptoms in sham, EAE^low^, and EAE^low^ + Ki16425 (15 and 30 mg/kg) groups were recorded daily (**A**) and scores from days 9–20 were summed (**B**). **C**, **D** At 19–20 days after immunization, lumbar spinal cords (n = 3) were obtained from each group and their lysates were analyzed by Western blot to determine protein expression levels of LPAR1 (**C**) and LPAR3 (**D**) followed by quantification. **E**, **F** At 19–20 days after immunization, cryosections (*n* = 3 per spinal cord) of lumbar spinal cords (*n* = 3 per group) were stained with LFB (left panel in **E**) and quantified (**F**). **G** Lysates from lumbar spinal cords were analyzed by Western blot to determine protein expression levels of MBP followed by quantification (**G**). **H** At 19–20 days after immunization, Ki16425 treatment was withdrawn. Bars = 100 µm. Data are expressed as mean scores of motor disability, demyelination score, or protein expression ± SEM (ANOVA test; ^#^*p* < 0.05 and ^##^*p* < 0.01 versus sham group; *p < 0.05 and **p < 0.01 versus EAE^low^ group)

To examine whether Ki16425, LPAR 1–3 antagonist, might have a beneficial or detrimental effect on motor disability of EAE mice, mice were immunized with MOG_35–55_ peptide to induce EAE with mild symptom (referred to as EAE^low^) and i.p. treated with Ki16425 at daily dose of 15 or 30 mg/kg or vehicle (5% DMSO/PBS) until the end of the experiment from the onset stage of symptom (day 9 after EAE^low^ induction) (Fig. 1A). Mice in the EAE^low^ group displayed mild motor disability (mean score, 1.31 ± 0.2) from the onset stage. However, treatment with 30 mg/kg of Ki16425 significantly deteriorated motor disability of EAE^low^ during the experimental period (Fig. 2A). From the onset day of motor disability to 19–20 days, maximum scores of motor disability (2.8 ± 0.4) and cumulative scores of motor disability (21.9 ± 1.8) in the 30 mg/kg Ki16425-treatment groups were significantly higher than those in the EAE^low^ group (1.5 ± 0.3 and 14.4 ± 2.0, respectively) (Fig. 2A, B). Protein expression levels of LPAR1 in spinal cords of EAE^low^ mice were slightly but not significantly enhanced than those in the sham group, whereas levels of LPAR3 were significantly increased. However, Ki16425 treatment reduced their protein expression levels in a dose-dependence manner (Fig. 2C, D). Since CNS demyelination is a typical histopathological feature of MS patients and EAE^low^ model [25, 26], whether worsened motor disability after Ki16425 treatment might have a significant relation with the level of CNS demyelination was then investigated. The 30 mg/kg Ki16425 treatment conspicuously increased levels of spinal demyelination (pale portion in white matter) after staining white matters of spinal cords with LFB dye on day 19–20 following EAE^low^ induction (Fig. 2E, F), in concordance with the pattern of results from MBP immunofluorescence staining (Fig. 2E right panel) and Western blot analyses (Fig. 2G). Additionally, we explored whether the withdrawal effect of Ki16425 might offset the deteriorating effect of Ki16425 in the same model. As expected, withdrawal of Ki16425 from day 18 after EAE^low^ induction (the highest score of motor disability: 1.8 ± 0.3 in 15 mg/kg Ki16425 and 2.3 ± 0.2 in 30 mg/kg Ki16425) perfectly neutralized it detrimental effects for motor disability (lowest score of motor disability: 1.2 ± 0.1 in 15 and 30 mg/kg) (Fig. 2H).

### Ki16425 enhances infiltration of immune cells such as CNS-resident microglia and blood-borne macrophages in the spinal cord of EAE^low^ mice

**Fig. 3:**
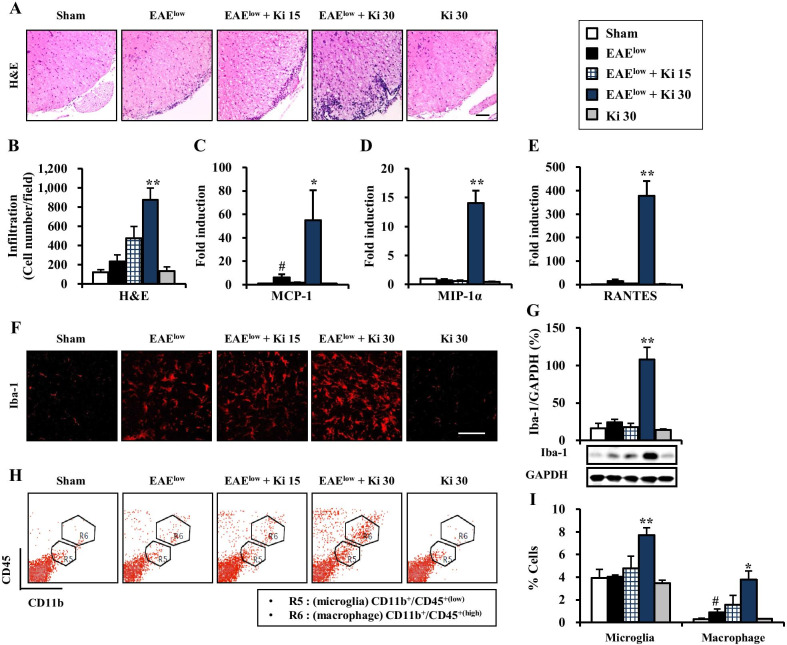
Effects of Ki16425 on immune cell infiltration and microglial activation in spinal cords of EAE^low^ mice. Lumbar spinal cords (*n* = 3 per group) were obtained from the sham, EAE^low^, EAE^low^ + Ki16425 (15 and 30 mg/kg), and Ki16425 (30 mg/kg) groups at day 19–20 post-immunization. **A**, **B** Cryosections (*n* = 3 per spinal cord) of lumbar spinal cord (*n* = 3) from each group were stained with hematoxylin and eosin dye (**A**) and quantified (**B**). **C**–**E** Lysates of lumbar spinal cords (*n* = 3) from each group were analyzed with real-time PCR to measure mRNA expression levels of MCP-1 (**C**), MIP-1α (**D**), and RANTES (**E**). **F**, **G** Cryosections (*n* = 3 per spinal cord) were subjected to immunofluorescent staining with anti-Iba-1 antiserum (**F**). Lysates were subjected to Western blot to measure Iba-1 protein expression followed by quantification (**G**). **H**, **I** Lumbar spinal cords (*n* = 3 per group) were cropped at 19–20 days after immunization to measure the level of infiltration of microglia and macrophages using flow cytometry. CD11b^+^ cells were divided into CD11b^+^/CD45^low^ cells (R5; microglia) and CD11b^+^/CD45^high^ cells (R6; macrophage) populations (**H**) and graphs (**I**) were made to show the percentages of each cell population. A total of 1 × 10^4^ events acquired within the combined gate based on FSC and SSC were used. Bars = 100 µm. Data are expressed as mean expressive value ± SEM (ANOVA test; ^#^*p* < 0.05 versus sham group; *p < 0.05 and **p < 0.01 versus EAE^low^ group)

To examine whether the deteriorated motor disability and spinal demyelination observed in Ki16425-treated EAE^low^ mice might have a connection with alleviation in CNS inflammation, the level of cellular infiltration in the spinal cord was determined by H&E staining (Fig. 3). The level of cellular infiltration was slightly enhanced in the white matter of the spinal cord of the EAE^low^ group compared to that of the sham group. However, this level was notably increased in 30 mg/kg Ki16425-treated EAE^low^ group (Fig. 3A, B). Cellular infiltration into the CNS lesion site is known to be associated with an increase of chemokine expression [2, 3, 6]. Thus, mRNA expression levels of representative chemokines (MCP-1, MIP-1α, and RANTES) in the spinal cord at 19–20 days after EAE^low^ induction were determined by real-time PCR analysis (Fig. 3C–E). 30 mg/kg Ki16425 treatment remarkably increased mRNA expression levels of MCP-1 (55. ± 25.6 folds), MIP-1α (14.1 ± 2.1 folds), and RANTES (377.7 ± 63.6 folds) in the spinal cord after EAE^low^ induction compared to that of the sham (vehicle-treated EAE^low^) group (Fig. 3C–E).

Cellular infiltrates into CNS can include CNS-resident microglia, blood-borne macrophages, T cells, and so on. Therefore, whether Ki16425 treatment affected their infiltration into demyelinated lesion was determined by immunohistochemistry. 30 mg/kg Ki16425 treatment remarkably increased the level of Iba-1 immunoreactivity in the spinal cord following EAE^low^ induction compared to that in the EAE^low^ group (Fig. 3F), corresponding to the upregulated protein expression of Iba-1 as shown by Western blot analysis (Fig. 3G). Since Iba-1 antibody recognizes both microglia and macrophages [27], differentiating both cell types based on immunostaining and Western blot has a limitation. Thus, both cells were further characterized by flow cytometry (Fig. 3H, I). Upon immunization, the percentage of CD11b^+^/CD45^+(low)^ cells (R5 rectangle in Fig. 3H) representing resident microglia [20–22, 24] increased to 4.0 ± 0.2% in spinal cords of the EAE^low^ group. Interestingly, this percentage was further increased to 7.7 ± 0.7% in spinal cords of Ki16425-treated group (Fig. 3H, I). The percentage of CD11b^+^/CD45^+(high)^ cells (R6 rectangle in Fig. 3H) representing macrophages [20–22, 24] was also increased to 0.9 ± 0.3% in spinal cords of EAE^low^ group. By the way, this percentage was further increased to 3.8 ± 0.7% in spinal cords of Ki16425-treated group (Fig. 3H, I).

### Ki16425 induces the hypertrophy of lymphatic organs and the CD4^+^ T cell infiltration in the spinal cords of EAE^low^ mice

**Fig. 4:**
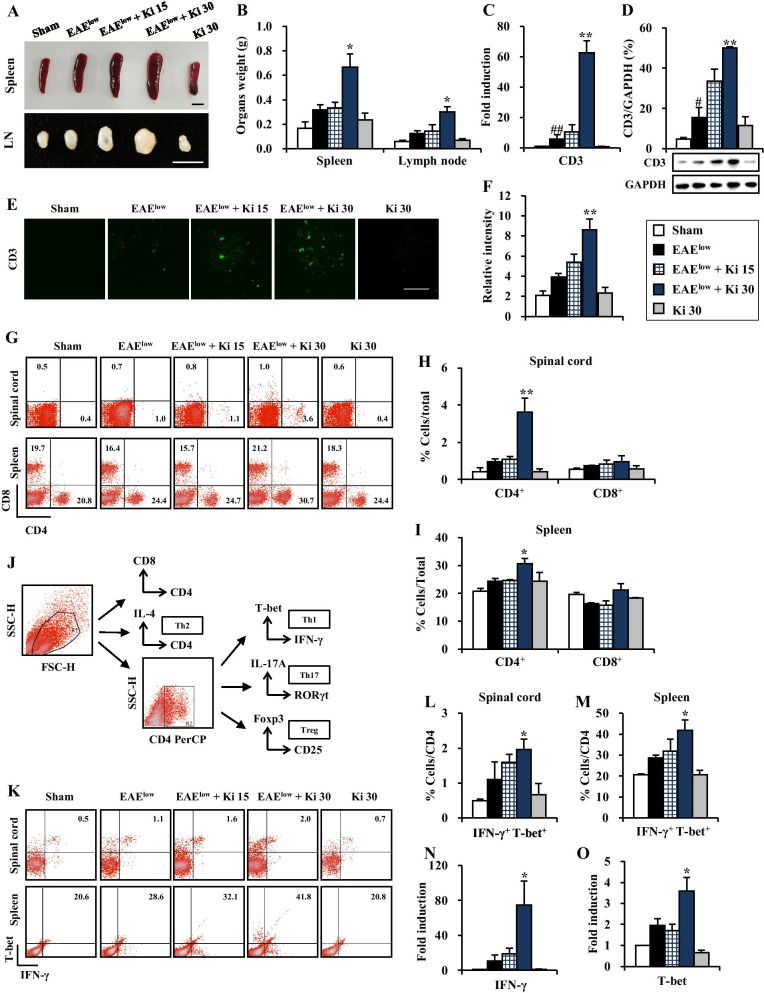

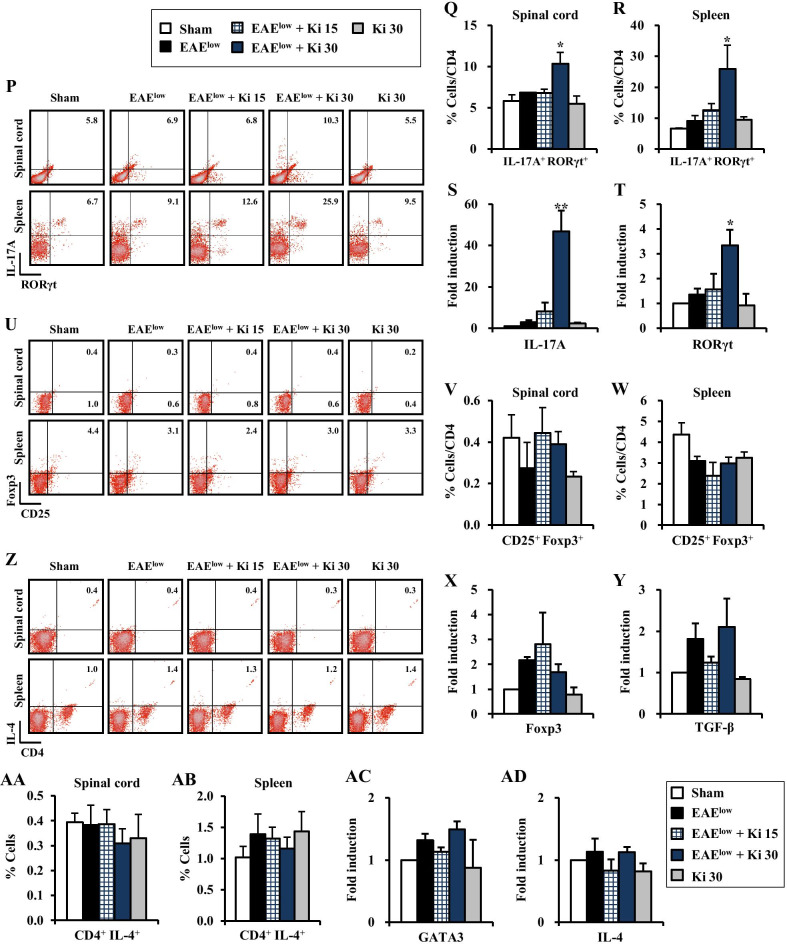
Effects of Ki16425 on hypertrophy of secondary lymphatic organs and population of CD4^+^, CD8^+^, Th1, Th2, Th17, and Treg cells in spinal cords or spleens of EAE^low^ mice. **A**, **B** Spleens and lymph nodes (*n* = 5 per group) were dissected (**A**) from sham, EAE^low^, EAE^low^ + Ki16425 (15 and 30 mg/kg), and Ki16425 (30 mg/kg) groups at day 19–20 post-immunization and weighed (**B**). **C**–**F** Lysates or cryosections of lumbar spinal cords (*n* = 3) from each group were used to analyze the degree of infiltration of T cells with CD3 mRNA level by real-time PCR (**C**), CD3 protein level by Western blot (**D**), and CD3 distribution by immunofluorescence stain (**E**, **F**). **G**–**I** Spinal cords and spleens (*n* = 3) from each group were used to investigate populations of CD4^+^ and CD8^+^ T cells by flow cytometry. Populations of CD4^+^ and CD8^+^ T cell were dotted (**G**) and displayed in graphs (**H**, **I**). **J** CD4^+^ subset gating scheme. To determine populations of CD4^+^ subset by flow cytometry, CD4^+^ T cells (1 × 10^4^) were first gated using FSC and SSC properties form spinal cords and spleens from each group. CD4^+^ T cells were used to analyze populations of Th1 (CD4^+^/IFN-γ^+^/T-bet^+^), Th17 (CD4^+^/IL-17A^+^/RORγt^+^), Treg (CD4^+^/CD25^+^/Foxp3^+^), and Th2 (CD4^+^/IL-4^+^) cells on CD4^+^ T cells. **K**–**M**, **P**–**R**, **U–W**, and **Z**–**AB** Populations of Th1, Th17, Treg, and Th2 cells were dotted (**K**, **P**, **U**, and **Z**, respectively) and displayed in graphs (**L**, **M**, **Q**, **R**, **V**, **W**, **AA**, and **AB**). **N**, **O**, **S**, **T**, **X**, **Y**, **AC**, and **AD** Lysate of lumbar spinal cords (*n* = 3) from each group were analyzed by real-time PCR to determine mRNA expression levels of IFN-γ (**N**), T-bet (**O**), IL-17A (**S**), RORγt (**T**), Foxp3 (**X**), TGF-ß (**Y**), GATA3 (**AC**), and IL-4 (**AD**). Quantified data were expressed as mean % cells or fold induction ± SEM. (ANOVA test; ^#^*p* < 0.05 and ^##^*p* < 0.01 versus sham group; *p < 0.05 and **p < 0.01 versus EAE^low^ group)

Secondary lymphoid organs such as spleen and lymph nodes might be hypertrophied in EAE murine model [28]. Thus, their weights were measured to compare their hypertrophy levels at 19–20 days following EAE^low^ induction. Weights of spleen and lymph nodes were slightly increased in the EAE^low^ group (0.32 ± 0.04 g and 0.12 ± 0.02 g, respectively), but they are not significant. However, their weights were further increased in the 30 mg/kg Ki16425-treated EAE^low^ group (0.67 ± 0.11 g and 0.30 ± 0.04 g, respectively; Fig. 4A, B). Since recruitment and infiltration of autoreactive T cells are major initiators and mediators of pathogenesis in MS and its animal models [1–3], the levels of T cell infiltration into spinal cords following EAE induction were measured by real-time PCR analysis, Western blot, and immunofluorescence stain for CD3, a marker for T cells [29]. The mRNA expression of CD3 was slightly enhanced in spinal cords of the EAE^low^ group at 19–20 days after EAE^low^ induction (5.8 ± 2.8 folds), whereas its expression level was more enhanced in spinal cords of 30 mg/kg Ki16425-treated EAE^low^ group (62.5 ± 8.2-fold) (Fig. 4C). Protein expression levels of CD3 were also slightly upregulated in spinal cords of the EAE^low^ group at 20 days after EAE^low^ induction (15.6 ± 4.9%), whereas its expression levels were more upregulated in spinal cords of 30 mg/kg Ki16425-treated EAE^low^ group (50.1 ± 0.5%) (Fig. 4D). Moreover, the expression pattern of CD3 immunoreactive cells (Fig. 4E, F) was in agreements with mRNA (Fig. 4C) and protein expression patterns of CD3 (Fig. 4D). By flow cytometry analysis, the percentage of CD4^+^ T cells was also slightly enhanced in spinal cords (1.0 ± 0.2%) and spleen (24.4 ± 1.0%) following EAE^low^ induction, but they are not significant. However, their increases were much more enhanced in the spinal cords (3.6 ± 0.7%) and spleen (30.7 ± 1.8%) of 30 mg/kg Ki16425-treated EAE^low^ group (Fig. 4G–I). As expected, the percentage of cytotoxic CD8^+^ T cells was not significantly affected by EAE^low^ induction or Ki16425 treatment (Fig. 4G–I).

### Ki16425 increases the percentages of Th1 and Th17 cells in the spinal cords of EAE^low^ mice

Naive CD4^+^ T cells are activated after interaction with antigen–major histocompatibility complex to differentiate into specific subtypes, such as Th1, Th2, Th17, and regulatory T (Treg) cells during T cell receptor (TCR) activation in a particular cytokine milieu, which are involved in autoimmunity and produce proinflammatory cytokines such as IFN-γ, IL-4, IL-17, and TGF-β, respectively [30, 31]. Therefore, subtypes of CD4^+^ T cells in spinal cord and spleen after EAE^low^ induction were discriminated. In the EAE^low^ group, percentages of Th1 (CD4^+^/IFN-γ^+^/T-bet^+^) cells was 1.1 ± 0.6% in the spinal cords and 28.6 ± 1.3% in the spleen. Interestingly, in the EAE^low^ + 30 mg/kg Ki16425 group, their percentages were more increased to 2.0 ± 0.3% in spinal cords and to 41.8 ± 5.1% in the spleen (Fig. 4K–M), corresponding to the pattern of mRNA expression of IFN-γ (an interleukin produced by Th1 cells) and T-bet (a transcription factor of Th1 cells) in spinal cords (Fig. 4N, O). In the EAE^low^ group, percentages of Th17 (CD4^+^/IL-17A^+^/RORγt^+^) cells was 6.9 ± 0.0% in the spinal cords and 9.1 ± 1.7% in the spleen. Interestingly, in the EAE^low^ + 30 mg/kg Ki16425 group, percentages of Th17 cells was more enhanced to 10.3 ± 1.4% in spinal cords and to 25.9 ± 7.7% in the spleen (Fig. 4P–R). In concordance with these results, mRNA expression levels of IL-17A (an interleukin produced by Th17 cells) and RORγt (a transcription factor of Th17 cells) were conspicuously enhanced in spinal cords of Ki16425-treated EAE^low^ group (Fig. 4S, T). However, percentages of Treg (CD4^+^/CD25^+^/Foxp3^+^) cells for maintaining tolerance to self-antigens and suppressing autoimmune responses were not significantly affected by EAE^low^ induction with Ki16425 treatment (Fig. 4U–W), corresponding to the pattern of mRNA expression of Foxp3 (a transcription factor of Treg cells) and TGF-β (an interleukin produced by Treg cells) in spinal cords (Fig. 4X, Y). Also, the percentage of Th2 (CD4^+^/IL-4^+^) cells was not significantly altered after EAE^low^ induction with Ki16425 treatment, in agreement with significantly unaltered mRNA expression levels of GATA3 (a transcription factor of Th2 cells) and IL-4 (an interleukin produced by Th2 cells) (Fig. 4Z–AD).

### Ki16425 stimulates BBB disruption in spinal cords of EAE^low^ mice

**Fig. 5:**
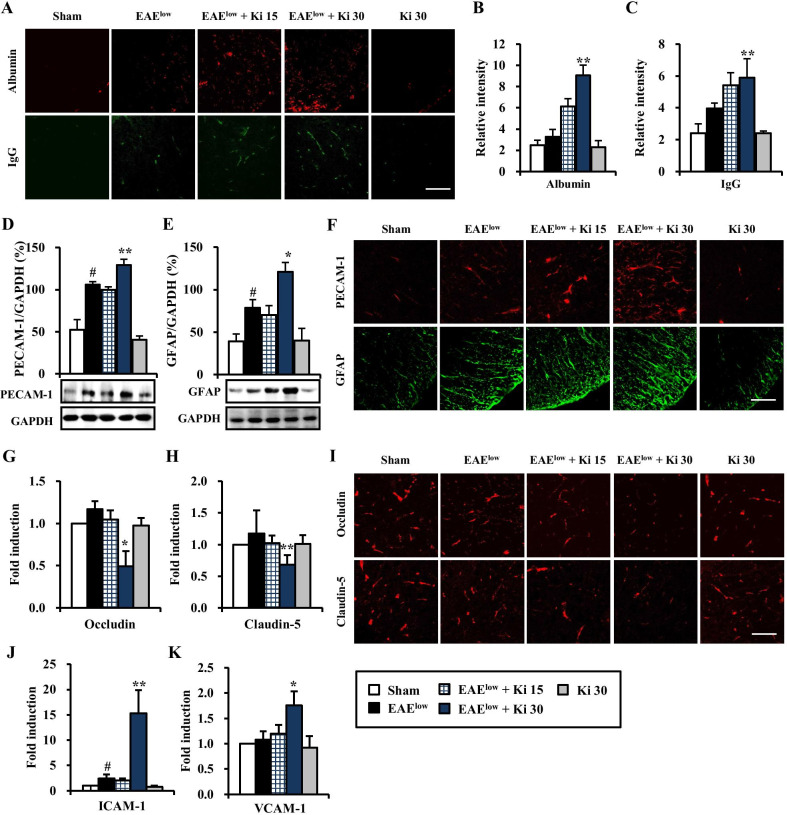
Effect of Ki16425 on BBB disruption in spinal cords of EAE^low^ mice. **A**–**K** Lumbar spinal cords (*n* = 3 per group) were dissected from the sham, EAE^low^, EAE^low^ + Ki16425 (15 and 30 mg/kg), and Ki16425 (30 mg/kg) groups at day 19–20 after immunization. **A**, **F**, and **I** Cryosections (*n* = 3 per spinal cord) were subjected to immunofluorescence staining with anti-albumin (upper panel in **A**), anti‐IgG (lower panel in **A**), anti-PECAM-1 (upper panel in **F**), anti‐GFAP (lower panel in **F**), anti-occludin (upper panel in **I**), or anti-claudin-5 antibody (lower panel in **I**). Their images were captured by confocal microscope and the levels of relative intensity were measured (**B** and **C**). **D** and **E** Lysates from lumbar spinal cords were analyzed by Western blot to investigate protein expression of PECAM-1 (**D**) and GFAP (**E**). **G**, **H**, **J**, and **K** The lysates (*n* = 3 per group) were analyzed by real-time PCR to measure the degrees of occludin (**G**) and claudin-5 (**H**) to represent tight junctions and ICAM-1 (**J**) and VCAM-1 (**K**) to represent cell adhesion molecules. Bar = 100 µm. Quantified data are expressed as mean protein level or fold induction ± SEM. (ANOVA test; ^#^*p* < 0.05 versus sham group; *p < 0.05 and **p < 0.01 versus EAE^low^ group

Disruption of the BBB is one of the major features in the progression of MS and EAE. Peripheral inflammatory cells and toxic molecules can migrate into the CNS via the damaged BBB, resulting in cerebral edema, demyelination, and neural cell death [4, 5]. Consequentially, we examined whether Ki16425 was involved in the maintenance of BBB integrity on day 19–20 after EAE^low^ induction. Levels of leakage of albumin and IgG by immunofluorescence staining were slightly increased in spinal cords of EAE^low^ group than those in the sham group. Their levels were much more increased in spinal cords of mice in the 30 mg/kg Ki16425-treated EAE^low^ groups (Fig. 5A–C). Protein expression levels of PECAM-1 (an important indicator of BBB dysfunction) and GFAP (a specific marker of astrocytes) were upregulated in spinal cords of EAE^low^ group than those in the sham group. Their expression levels were much more increased in spinal cords of mice in the 30 mg/kg Ki16425-treated EAE^low^ group (Fig. 5D, E). In accordance with these results, PECAM-1 and GFAP-positive immunofluorescence signals were also clearly enhanced in spinal cords of mice in the 30 mg/kg Ki16425-treated EAE^low^ group (Fig. 5F). Tight junctions are specialized cell–cell adhesion structures and critical components of the BBB. They might be abnormally distributed in CNS tissues of MS patients and EAE mice [4, 5, 32–34]. Thus, we further investigated whether Ki16425 treatment exerted beneficial or detrimental effects on the expression of transmembrane molecules of tight junctions in spinal cords of mice on day 20 after EAE^low^ induction by real-time PCR analysis (Fig. 5G, H). After treatment with Ki16425 at 30 mg/kg, mRNA expression levels of occluding and claudin-5 in spinal cords of mice in the EAE^low^ group were reduced (0.5-fold and 0.7-fold, respectively) compared to those in the sham group (1.0-fold) or the EAE^low^ group (1.2-fold) (Fig. 5G, H). These results coincided with patterns of immunofluorescence expression of the occluding and claudin-5 (Fig. 5I). Since cellular adhesion molecules such as ICAM-1 and VCAM-1 are involved in the adhesion of peripheral inflammatory cells such as lymphocytes and macrophages to endothelial cells in lesion area during MS and EAE^low^, we investigated the effect of Ki16425 treatment on their expression by real-time PCR analysis (Fig. 5J, K). After Ki16425 treatment, mRNA expression levels of ICAM-1 and VCAM-1 in spinal cords after EAE^low^ induction were significantly upregulated (15.3-fold and 1.8-fold, respectively) compared to those in the EAE^low^ group (2.4-fold and 1.1-fold, respectively) (Fig. 5J, K).

### Ki16425 induces proinflammatory cytokine milieu and oxidative stress in spinal cords after EAE^low^ induction

**Fig. 6:**
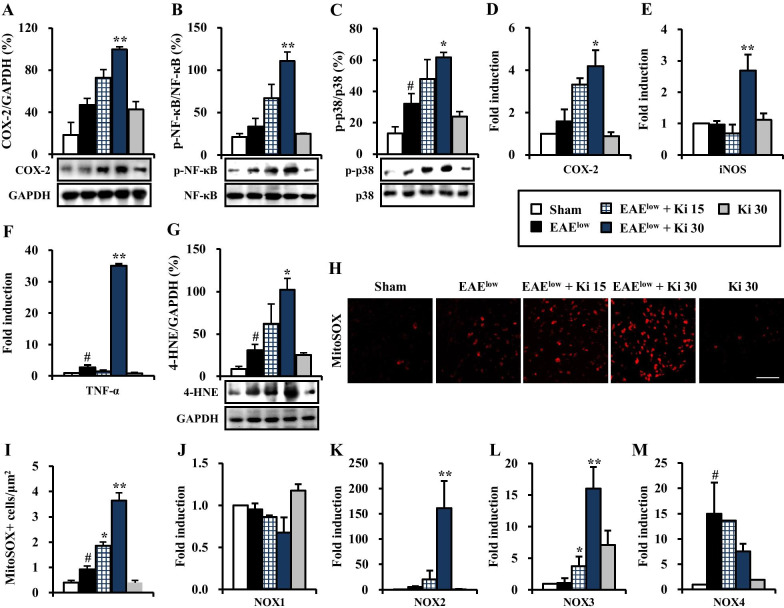
Effect of Ki16425 on proinflammatory cytokine milieu and oxidative stress in spinal cords after EAE^low^ induction. **A**–**M** Protein lysates and total RNA were isolated from the lumbar spinal cords (*n* = 3 per group) of the sham, EAE^low^, EAE^low^ + Ki16425 (15 and 30 mg/kg), and Ki16425 (30 mg/kg) groups at day 19–20 after immunization. **A**–**C** The lysates (*n* = 3 per group) were analyzed by Western blot using COX-2 (**A**), p-NF-κB (**B**), and p-p38 MAPK (**C**) antibodies to represent inflammatory signaling pathways and the results were quantified (**A**–**C**). **D**–**F** Total RNA (*n* = 3 per group) was analyzed by real-time PCR to measure mRNA expression of COX-2 (**D**), iNOS (**E**), and TNF-α (**F**) as representative cytokines. **G**–**I** Lysates of lumbar spinal cords (*n* = 3 per group) from sham, EAE^low^, EAE^low^ + Ki16425 (15 and 30 mg/kg), and Ki16425 (30 mg/kg) groups at day 19–20 after immunization were analyzed by Western blot using 4-HNE antibody followed by quantification (**G**). Cryosections (*n* = 3 per spinal cord) of lumbar spinal cords (*n* = 3 per group) from each group were subjected to MitoSOX™ assay to measure mitochondrial superoxide level (**H**) followed by quantification (**I**). **J**–**M** Lumbar spinal cords (*n* = 3) from each group were analyzed by real-time PCR to investigate mRNA expression levels of NOX1 (**J**), NOX-2 (**K**), NOX3 (**L**), and NOX4 (**M**). Bar = 100 µm. Data are expressed as mean value, fold induction, or cell number ± SEM (ANOVA test; ^#^*p* < 0.05 versus sham group; *p < 0.05 and **p < 0.01 versus EAE^low^ group)

Since inflammation is critically involved in pathological mechanism underlying MS and EAE [35, 36], we tested the effect of Ki16425 on inflammatory response in spinal cords at day 19–20 after EAE^low^ induction (Fig. 6). The severity of inflammatory response was compared based on expression levels of COX-2, p-p38 MAPK, and p-NF-κB/p65 known as central mechanisms of inflammation. Expressions of COX-2, p-p38 MAPK, and p-NF-kB/p65 showed a quietly enhancing pattern in spinal cords of EAE^low^ group (46.6%, 33.2%, and 32.1%, respectively) compared to those in the sham group (18.5%, 20.9%, and 13.0%, respectively). However, their expression levels were notably enhanced by 30 mg/kg Ki16425 treatment (99.4%, 110.4%, and 61.7%, respectively) compared to vehicle treatment (Fig. 6A–C). In accordance with these results, mRNA expression levels of representative inflammatory enzymes COX-2 and iNOS and a representative proinflammatory cytokine TNF-α were also significantly increased by 30 mg/kg Ki16425 treatment (Fig. 6D–F). Ki16425 itself did not significantly regulate the expression of inflammatory enzymes or proinflammatory cytokine and the phosphorylation of p38 MAPK and NF-κB pathways (Fig. 6A–F).

Excessive oxidative stress plays a critical role in various inflammatory mechanisms underlying MS and EAE [7, 9, 37, 38]. Thus, we tested the effect of Ki16425 on oxidative stress in the spinal cords of mice on day 19–20 after EAE^low^ induction (Fig. 6G–M). The level of oxidative stress was measured with 4-hydroxynonenal (4-HNE) widely accepted as a stable marker for oxidative stress [39]. Protein expression levels of 4-HNE was in slightly increasing pattern in spinal cords of the EAE^low^ group (31.0%) compared to those in the sham group (8.8%), while they were strikingly upregulated in 30 mg/kg Ki16425-treated EAE^low^ group (102.1%) (Fig. 6G). Mitochondrial superoxide level was also analyzed by MitoSOX™ Red assay. The median fluorescence intensity was significantly increased in spinal cords of the EAE^low^ group (0.9 cells/μm^2^) compared to that in the sham group (0.4 cells/μm^2^). However, the intensity was much more increased after Ki16425 treatment (3.7 cells/μm^2^) (Fig. 6H, I). Oxidation status (ROS level) in the spinal cord was determined based on NADPH oxidase (NOX) expression and NADPH activity. Although mRNA levels of NOX1–3 were not significantly changed in spinal cords after treatment with Ki16425 compared to those in the EAE^low^ group without such treatment, mRNA levels of NOX2 and NOX3 were significantly increased by treatment with Ki16425 at 15 or 30 mg/kg compared to those in the EAE^low^ group without such treatment (Fig. 6J–L). In spinal cords of the EAE^low^ group without treatment with Ki6425, NOX4 mRNA levels were significantly enhanced. However, NOX4 mRNA levels were not significantly altered by Ki16425 treatment (Fig. 6M). These results indicate that Ki16425 treatment might deteriorate EAE by activating NOX2/3 signaling.

### LPAR1/2 agonist, 1-oleoyl-LPA, mitigates motor disability and main pathological features of EAE^high^ mice

**Fig. 7:**
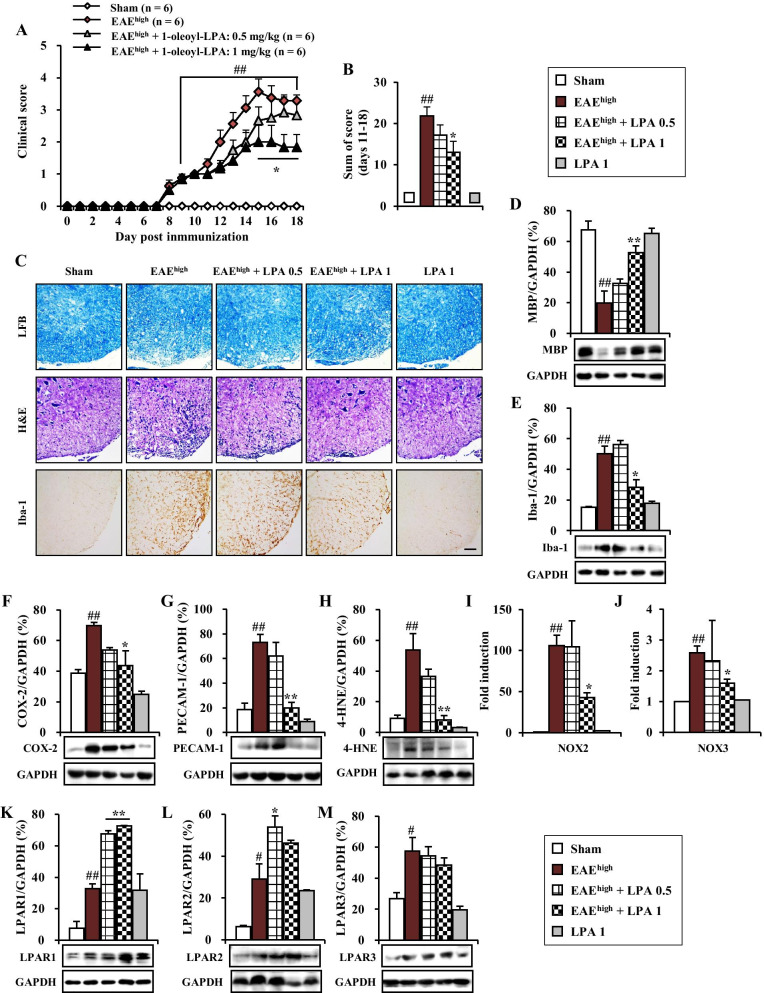
Effects of 1-oleoyl-LPA, an agonist of LPAR1/2, on neurological symptoms and main pathophysiological features in spinal cords of mice after EAE^high^ induction. **A**, **B** Following immunization, neurological symptoms in sham, EAE^high^, and EAE^high^ + 1-oleoyl-LPA (0.5 and 1 mg/kg) groups were measured daily (**A**) and scores from days 11–18 were summed (**B**). **C** At 19 days after immunization, cryosections (*n* = 3 per spinal cord) of lumbar spinal cords (*n* = 3 per group) were stained with LFB (upper panel), H&E stain (middle panel), or immunochemical stained with Iba-1 antibody (lower panel). **D**–**H**, **K**–**M** At day 19, lysates of lumbar spinal cords (*n* = 3) from each group were analyzed by Western blot to measure protein expression levels of MBP (**D**), Iba-1 (**E**), COX-2 (**F**), PECAM-1 (**G**), 4-HNE (**H**), LPAR1 (**K**), LPAR2 (**L**), and LPAR3 (**M**). **I**, **J** Total RNA (*n* = 3 per group) was extracted and used for real-time PCR to measure mRNA expression levels of NOX2 (**I**) and NOX-3 (**J**). Data are expressed as mean expressive value ± SEM (ANOVA test; ^#^*p* < 0.05 and ^##^*p* < 0.01 versus sham group; *p < 0.05 and **p < 0.01 versus EAE^high^ group)

In the current study, LPAR1–3 antagonist, Ki16425, remarkably worsened motor disability of EAE^low^ mice (Fig. 2A, B), in accordance with increased demyelination (Fig. 2E–G), enhanced inflammatory response (Figs. 3, 4, and 6), amplified ROS pathway (Fig. 6), and worsened BBB integrity (Fig. 5) in spinal cords after EAE^low^ induction. These findings suggest that activating the LPA signaling pathway might have beneficial effects on EAE^low^. To demonstrate this possibility, 1-oleoyl-LPA, a LPAR1/2 agonist, was i.p. injected to mice for 10 days from the onset stage of EAE^high^ symptom (day 8 after EAE^high^ induction with a mean score of 0.5 ± 0.2). As expected, 1-oleoyl-LPA mitigated motor disability of EAE^high^ (Fig. 7A, B) along with a reduction of demyelination (Fig. 7C upper panel and D), cellular infiltration (Fig. 7C middle panel), microglial activation (Fig. 7C lower panel and E), expression of a representative inflammatory enzyme COX-2 (Fig. 7F), protein expression of a representative BBB marker PECAM-1 (Fig. 7G), protein expression of a representative oxidative stress marker 4-HNE (Fig. 7H), and mRNA expression of NOX2 and NOX3 (Fig. 7I, J). Treatment with 1-oleoyl-LPA also enhanced protein expression levels of LPAR1 and LPAR2, but not LPAR3 in spinal cord of EAE^high^ group. In addition, protein expression levels of LPAR1, LPAR2, and LPAR3 were enhanced in in spinal cords of the EAE^high^ group compared to those in the sham group (Fig. 7K–M).

These results suggest that motor disability and main pathological events of EAE^high^ mice can be neutralized by pre-stimulating the LPAR signaling pathway with LPAR1/2 agonist.

## Discussion

Here, we demonstrated that antagonizing LPAR 1–3 with Ki16425 deteriorated specific motor disability and spinal demyelination after EAE induction, corresponding to increased cellular infiltrates (such as microglia, macrophage, Th1, and Th17 cells) and worsened BBB integrity. The mechanism underlying the deteriorated EAE was excessive oxidative stress via NOX2 and NOX3. Interestingly, LPAR1/2 agonist 1-oleoyl-LPA improved neurological symptoms and representative pathophysiological characteristics of EAE. These results provide new mechanistic insights into how LPA 1–3 signaling contributes to EAE pathophysiology. Taken together, our findings suggest that agents that can regulate LPAR 1–3 might be used as therapeutics for treating MS.

LPARs are differentially expressed on most cell types within central and peripheral nervous tissues. They preferentially bind to saturated, monounsaturated, and polyunsaturated LPAs [14]. Signal transduction through LPARs has been functionally linked to many neural processes, including cell proliferation, cell survival, apoptosis, morphological change, cell migration, and the production of other lipids such as prostaglandins through arachidonic acid conversion by cyclooxygenase-2 [14]. Therefore, LPARs have been considered as novel targets in lipidomic-based therapeutics for neurological disorders [16]. Many neurological disorders frequently accompany demyelination-associated signs and symptoms such as neuropathic pain, demyelinating neuropathies, and MS [20–22]. Loss of LPAR1 can impair oligodendrocyte differentiation and myelination due to impaired intracellular transport of the proteolipid protein (PLP)/DM20 myelin protein in the mouse cerebral cortex [40]. LPA1-null mutant mice have shown delayed Schwann cell-to-axon segregation, polyaxonal myelination by single Schwann cell, and thinner myelin sheaths via heterotrimeric G-alpha protein, Gαi, and small GTPase, Rac1 signaling [15]. LPA2-deficient mice have shown enhanced motor skills and myelin sparing after spinal cord injury related to oligodendrocyte cell death by activating microglial LPA2 [41]. LPA signaling is involved in various neurological diseases such as Alzheimer’s disease and traumatic brain injury [42, 43]. In traumatic brain injury, LPA activity is increased due to upregulated expression of LPAR1, LPAR2, and LPAR [42, 43]. In this study, protein expression levels of LPAR1–3 were increased in spinal cords of EAE^low^ and EAE^high^ mice (Figs. 2 and 7). Such an increase of LPA activity seems to be involved in early pathologic processes such as neurite retraction, reactive gliosis, inflammation, and cell death after trauma [42]. These reports strongly suggest that investigating new signaling mechanisms in these disorders might be critical in the development of therapeutics to stimulate spontaneous remyelination and subsequent functional recovery. In the present study, treatment with Ki16425, an LPAR1–3 antagonist, impaired motor disability and spinal demyelination after EAE^low^ induction (Figs. 2 and 3), whereas treatment with 1-oleoyl-LPA, an LPAR1/2 agonist, mitigated them after EAE^high^ induction (Fig. 7). These results indicate that LPA signaling via LPARs, specifically LPAR 1–3, might play a pivotal role in MS pathology.

Levels of resident microglia activation and infiltration of monocyte-derived immune cells to the CNS are associated with neurodegeneration in both MS and EAE [4]. Infiltrated immune cells are important contributors to the local chemical environment, releasing either anti-inflammatory growth factors or proinflammatory cytokines depending on their activation states. However, whether they have beneficial of detrimental roles remains controversial [4]. BV-2 cells express LPAR 2, 3, 5, and 6, whereas primary murine microglia express LPAR 1, 2, 4, 5, and 6 [44]. It has been shown that LPAR1 knockdown in the brain with its specific shRNA lentivirus can attenuate sepsis-induced microglia activation, morphological transformation, and proliferation, in agreement with the downregulation of TNF-α production by activating ERK1/2 in the brain and LPS-stimulated cells [45]. On the other hand, LPAR1–3 antagonist, Ki16425, reduced numbers and soma sizes of activated microglia. It also reduced microglial proliferation, in correspondence with reduced mRNA expression levels of proinflammatory cytokines and suppressed NF-κB activation in the ischemic brain. Particularly, these LPAR1-derived proinflammatory responses have appeared in activated microglia because NF-κB activation occurs mainly in activated microglia [46]. LPAR2 is constitutively expressed in the spinal cord parenchyma. Its transcripts are upregulated after spinal cord injury, in part, by microglial cells [41]. The demyelinating lesion triggered by intraspinal injection of LPA into the undamaged spinal cord was markedly reduced in the absence of LPAR2 [41]. LPAR2-deficient mice have shown enhanced locomotor skills and myelin sparing after spinal cord injury [41]. Thus, these previous reports suggest that LPAR1–3 has a novel function in microglial activation and that its mechanism could be involved in the pathogenesis of diverse neurological diseases related to microglial activation. Our previously study has shown that gintonin, a ginseng-derived lysophosphatidic acid receptor ligand, can reduce 3-nitropropionic acid-induced striatal toxicity through its antioxidant and anti-inflammatory activities. It downregulated microglial activation through LPA, whereas LPAR1–3 antagonist, Ki16425, neutralized gintonin’s beneficial effects [47]. In the present study, LPAR 1–3 antagonist, Ki16425, also increased microglial activation and infiltration of peripheral immune cells (macrophages) to demyelinating lesion following EAE induction (Figs. 2, 3, 4, 5 and 6), whereas LPAR 1/2 agonist inhibited them after EAE^high^ induction (Fig. 7). Our findings suggest that LPAR 1–3 might play a critical role in the EAE pathology via microglial activation and peripheral immune cell infiltration into lesion.

During MS and EAE process, naive T cells primed by antigen presenting cells such as microglia, macrophages, and dendritic cells can differentiate into Th1, Th2, Th17, or Treg cells depending on the cytokine environment [30]. Up to now, the role of LPA signaling in T cell differentiation is clearly unknown. In the current study, mild EAE did not significantly change the size of the spleen and the lymph nodes, the population (number) of CD4 cells or its major subsets, or the population of CD8 T cells in the spleen following EAE^low^ induction (Fig. 4). However, LPAR1–3 antagonist, Ki16425, clearly increased the size of the spleen and the lymph nodes and the population of CD4, Th1, and Th17 cells in the spleen associated with deteriorated EAE^low^ symptoms and pathological features. However, LPAR1–3 antagonist did not significantly influence the population of CD8, Th2, or Treg cells in the spleen (Fig. 4). In the MS and EAE, peripheral autoreactive T cells can migrate across the disrupted BBB, attack myelin antigens, and induce demyelination in the CNS [5]. Although the migration of autoreactive T cells is mediated by multi-step process of lymphocyte diapedesis through the BBB [5, 30], the role of LPA signaling in the process is largely unknown. LPA and LPA-generating enzyme autotaxin are constitutively expressed at high endothelial venules of lymph nodes. They are implicated in lymphocyte trafficking and the regulation of lymphocyte entry into lymph nodes [48]. LPA signaling mediates the recruitment of leukocytes including CD3 T cells into unprimed and TNF-α-primed air pouches in a murine air pouch model of inflammation [49]. LPAR5 is an inhibitory receptor that suppresses CD8 T cell cytotoxic function via disruption of early TCR signaling [50]. These reports strongly suggest that LPA signaling might have a critical role in T cell migration into demyelinating lesion of EAE. In the present study, mild EAE did not significantly increase the population of CD3^+^ T cell or major subsets of CD4 T cell in the spinal cord after EAE^low^ induction (Fig. 4). LPAR1–3 antagonist Ki16425 clearly increased the population of CD3 (T), CD4 (Th), Th1, and Th17 cells in the spinal cord associated with deteriorated EAE^low^ symptoms and pathological features (Fig. 4). However, LPAR1–3 antagonist Ki16425 did not significantly influence the population of CD8 (Tc), Th2, or Treg cells (Fig. 4). Such detrimental effect of LPAR1–3 antagonist Ki16425 could be supported by a previous similar report showing that LPAR2-deficiency mice induced more T cells trafficked from the spleen to the spinal cord, leading to a defect in lymphocyte homing which was reflected by impaired clinical scores and stronger activation of microglia in the grey matter of spinal cords of EAE mice [18]. Taken together, our findings suggest that LPA signaling via LPAR1–3 might have pivotal role in T cell differentiation in the secondary lymphatic organs and T cell migration into CNS after EAE induction.

The BBB consists of endothelial cells, pericytes, basal membrane, and foot process of astrocytes. It acts as structural and functional barrier to the crossing of peripheral immune cells (macrophages and T cells) into the CNS in vivo or cultured astrocytes expressing Lpar1–5 [51, 52]. The LPA1–3 antagonist Ki16425 has abolished LPA-induced vasorelaxation [53]. Cultured endothelial cells are known to express LPAR1–6 [54, 55]. LPA signaling can promote the survival and proliferation of endothelial cells from a variety of sources [56], including brain microvascular bEND.3 cells [54]. These reports suggest that LPAR antagonist might exert a negative effect on BBB maintenance. Here, we investigated the effect of LPAR1–3 antagonist Ki16425 on BBB integrity and permeability. LPAR1–3 antagonist Ki16425 enhanced levels of leakage of albumin and IgG by immunofluorescence staining in spinal cords of EAE^low^ mice (Fig. 5). And Ki16425 upregulated protein expression levels of GFAP and PECAM as well as mRNA expression levels of ICAM-1 and VCAM-1 in spinal cords of EAE^low^ mice (Fig. 5), in agreement with impaired motor disability of EAE^low^ (Fig. 2). These results suggest that LPAR1–3 antagonist, Ki16425, might deteriorate EAE symptom associated with impaired BBB disruption caused by excessive astrocytic activation and increased expression levels of ICAM-1 and VCAM-1 in the spinal cord (Fig. 5).

Pathologically, NOX produces an excessive amount of ROS including hydrogen peroxide (H_2_O_2_), superoxide (O^2•−^), and hydroxyl (OH^•^) radicals [9]. NOX2, NOX3, and NOX4 are the most prominently expressed NOX isotypes in the CNS. However, cellular and temporal expression profiles of these isotypes in injured and non-injured CNS are currently unclear [57]. In the MS and EAE, excessive ROS production overwhelms antioxidant defenses and induces oxidative damage (e.g., lipid peroxidation, protein nitration) in endothelial cells of the BBB and the myelin sheath, thereby propagating neurodegeneration [7, 8]. Activated microglia and infiltrated macrophages are responsible for ROS production in CNS lesions through upregulation of NOX2 [7, 8]. Isolated microglia from NOX2 knock-out mice show reduced oxidative stress-induced toxicity to oligodendrocytes. In addition, the mice are more resistant to EAE [9]. NOX3 is expressed in neurons in the inner ear. Reduction of NOX3 exerts a protective effect in cochlear injury by reducing the level of oxidative stress [9]. On the other hand, LPAR1 inhibitor AM095 treatment inhibits LPA-induced ROS production and NOX expression as well as LPA-induced toll-like receptor 4 expression in mesangial cells and in the kidney of streptozotocin-induced diabetic mice [58]. In addition, AM095 treatment suppressed LPA-induced proinflammatory cytokines through downregulation of phosphorylated NF-κBp65 and c-Jun N-terminal kinases in vitro and in the kidney of streptozotocin-induced diabetic mice [58]. LPA signaling through LPAR3 increased expression levels of antioxidant enzymes, consequently inhibiting ROS accumulation and ameliorating cell senescence. Moreover, in a zebrafish model, LPA3 deficiency was sufficient to cause premature aging phenotypes in multiple organs as well as a shorter lifespan [59]. These results suggest that LPA or LPAR subtypes might exert significant positive or detrimental effects on neurodegeneration. Thus, we investigated the effect of LPAR1–3 antagonist, Ki16425, on oxidative stress after EAE^low^ induction in the present study. LPAR1–3 antagonist Ki16425 significantly increased protein expression levels of 4-HNE, mRNA expression levels of NOX2 and NOX3, and NADPH activities in spinal cords of EAE^low^ mice compared to those in the EAE^low^ group associated with the enhanced microglial activation and the increased microphage infiltration (Figs. 2, 3, 4, 5 and 6). However, LPAR1/2 agonist 1-oleoyl-LPA significantly inhibited expression levels of ROS-associated markers in spinal cords of EAE^high^ mice (Fig. 7). These results indicate that LPAR1–3 antagonist, Ki16425, may induce oxidative stress via activation of NOX2 and NOX3 and that oxidative stress might lead to deterioration of EAE symptoms. Taken together, our findings indicate that regulation of NOX2 and NOX3 via LPAR 1–3 is a key contributor to MS and EAE.

## Conclusions

Ki16425, an antagonist of LPAR1–3, worsened EAE symptoms along with enhanced demyelination, inflammation, cellular infiltration, and BBB disruption caused by overproduction of ROS via NOX2 and NOX3, whereas 1-oleoyl-LPA, an agonist of LPAR1/2, alleviated them. These results suggest that functional activity of LPA signaling through LPAR1–3 might contribute to the pathophysiology of MS. Thus LPAR1–3 signaling might be a target to develop specific treatment for MS.

## Data Availability

All data generated or analyzed during this study are included in this published article and its additional files.

## Abbreviations

4-HNE: 4*-*Hydroxynonenal
BBB: Blood–brain barrier
CNS: Central nervous system
COX-2: Cyclooxygenase-2
EAE: Experimental autoimmune encephalomyelitis
FITC: Fluorescein-isothiocyanate
FSC: Forward scatter
GAPDH: Glyceraldehyde-3-phosphate dehydrogenase
GFAP: Glial fibrillary acidic protein
H&E: Hematoxylin and eosin
Iba-1: Ionized calcium binding adaptor molecule-1
ICAM-1: Intercellular adhesion molecule-1
IFN-γ: Interferon-gamma
IL: Interleukin
LFB: Luxol fast blue
LPARs: Lysophosphatidic acid receptors
MBP: Myelin basic protein
MS: Multiple sclerosis
NADPH: Nicotinamide adenine dinucleotide phosphate
NOX: NADPH oxidases
PECAM-1: Platelet endothelial cell adhesion molecule-1
NF-κB: Nuclear factor-kappa B
MCP-1: Monocyte chemoattractant protein-1
MIP-1α: Macrophage inflammatory protein-1α
RANTES: Regulated upon activation, normal T cell expressed and presumably secreted
ROS: Reactive oxygen species
SSC: Side scatter
TGF-ß: Transforming growth factor-ß
TNF-α: Tumor necrosis factor-α
Treg: Regulatory T
VCAM-1: Vascular cell adhesion molecule-1
